# Platelet-Rich Fibrin Used in Regenerative Endodontics and Dentistry: Current Uses, Limitations, and Future Recommendations for Application

**DOI:** 10.1155/2021/4514598

**Published:** 2021-12-15

**Authors:** Sohaib Arshad, Fatima Tehreem, Muhammad Rehab khan, Fatima Ahmed, Anand Marya, Mohmed Isaqali Karobari

**Affiliations:** ^1^Periodontics Unit, School of Dental Sciences, Health Campus, Universiti Sains Malaysia, Kubang Kerian, Kota Bharu 16150, Kelantan, Malaysia; ^2^Fatima Memorial Hospital College of Medicine and Dentistry, Shadman, Lahore, Pakistan; ^3^Department of Orthodontics, University of Puthisastra, Phnom Penh 12211, Cambodia; ^4^Department of Orthodontics, Saveetha Dental College & Hospitals, Saveetha Institute of Medical and Technical Sciences University, Chennai 600077, Tamil Nadu, India; ^5^Conservative Dentistry Unit, School of Dental Sciences, Universiti Sains Malaysia, Health Campus, Kubang Kerian, Kota Bharu 16150, Kelantan, Malaysia; ^6^Department of Conservative Dentistry & Endodontics, Saveetha Dental College & Hospitals, Saveetha Institute of Medical and Technical Sciences University, Chennai 600077, Tamil Nadu, India

## Abstract

Regenerative endodontics has introduced numerous procedures such as pulp implantation, revascularization, and postnatal stem cell therapy. Revascularization has been successfully implemented clinically nowadays, thus providing dentists with outrageous results. Platelet-rich fibrin (PRF) used either alone or along with bone graft promotes bone growth and vascularization. This matrix promotes migration, cell attachment, and proliferation of osteoblast that leads to bone formation. PRF consists of a packed fibrin complex consisting of leukocytes, cytokines, and glycoproteins such as thrombospondin. The usage of PRF has reported high success rates in surgical cases such as sinus lift procedures, healing of extraction sockets, and management of periapical abscesses. Compared to platelet-rich plasma, PRF is more economical, easy to prepare, and feasible to use in daily clinical practices. Revascularization compromised the induction of a blood clot into the root canal space, which emerged as a clinical triumph. This further led to platelet concentrates as an autologous scaffold on which revascularization could occur. The applications of PRF in regenerative endodontics are numerous, such as an agent for repairing iatrogenic perforation of the pulpal floor and for the revascularization of immature permanent teeth with necrotic pulps. It acts as a matrix for tissue ingrowth. Evidence of progressive thickening of dentinal walls, root lengthening, regression in the periapical lesion, and apical closure was reported. Further studies are needed to clarify the precise mechanism of action of PRF for dental pulp regeneration both in vitro and in vivo. The current review aims at the present uses of PRF in regenerative endodontics dentistry and its application with future recommendations and limitations.

## 1. Introduction

Procedures that involve the usage of materials that encourage healing and repair of the pulp dentin complex after restoring the infectious or diseased tooth tissue are known as regenerative procedures [1]. According to the concepts of regenerative therapy in dentistry, this procedure can turn a dead tooth into a vital one. Regenerative endodontics has introduced numerous procedures such as pulp implantation, revascularization, and postnatal stem cell therapy. Revascularization has been successfully implemented clinically nowadays, thus providing dentists with outrageous results [2]. Due to the increased rate of root canal treatment failure and posttreatment complications, regenerative endodontics plays an essential role in overcoming these complications. The goal is to prevent aggressive invasive instrumentation and radiographic exposure [3]. This is performed by the reimposition of *β* and T lymphocytes that aid in defense against the pathogens leading to pulp damage. The canal is completely sealed, and the vitality of the tooth is sustained, leading to the prevention of tooth fracture and periapical reinfection [4].

Healing is known to be a demand in surgical procedures, and it is achieved by a series of events: cellular organization, chemical signals, and extracellular matrix for tissue repair [5]. Platelet-rich fibrin used either alone or along with bone graft promotes bone growth and vascularization. This matrix promotes migration, cell attachment, and proliferation of osteoblast that leads to bone formation [6]. Cytokines released by PRF play a significant role in blood vessel formation and immune system stimulation to fight foreign pathogens [7]. Studies claim that PRF that is prepared using low centrifugal forces leads to the effective concentration of leukocytes and growth factors related to those prepared at high centrifugal forces [8]. A study has proved the enhanced results of PRF consumption along with iliac crest bone graft in patients with cleft alveolar ridge defects; relatively, in cases where iliac crest graft alone was used, results were unsatisfactory [9].

Similarly, an orthodontic surgical case was pursued using PRF, cancellous bone allograft, bovine bone matrix, and metronidazole, leading to complete healing with no complication [10]. Results obtained from studies have shown that when PRF is combined with the biomaterials, the respective substitute's revitalization power increases and is more suitable and acceptable for the defected tissue space. PRF develops the cell-to-cell interaction; thus, proper incorporation of biomaterial is attained [11]. The current review aims at the uses of PRF in regenerative endodontics and dentistry and its applications with future application and limitations.

## 2. Materials and Composition

**Figure 1:**
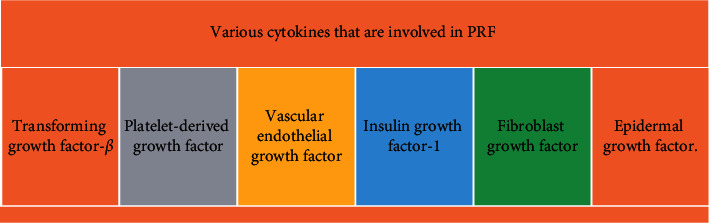
The various cytokines that are involved in PRF.

Being an unnatural biomechanical complex, platelet-rich fibrin (PRF) is synthesized by homologized plasma. This is performed by the formation of a fibrin clot which occurs during centrifugation of human-derived blood. This clot later contains a copious number of cytokines, growth factors, and platelets resulting from a polymerization reaction; apart from this, there is no foreign enzyme or anticoagulant needed for its production. Transforming growth factor-*β* (TGF-*β*), platelet-derived growth factor (PDGF), vascular endothelial growth factor (VEGF), insulin growth factor-1 (IGF-1), fibroblast growth factor (FGF), and epidermal growth factor (EGF) are the cytokines present in PRF which contribute in osteoblast proliferation, angiogenesis, wound healing, and collagen formation [5], Figure 1.

## 3. Classification of Plasma-Rich Fibrin

**Figure 2:**
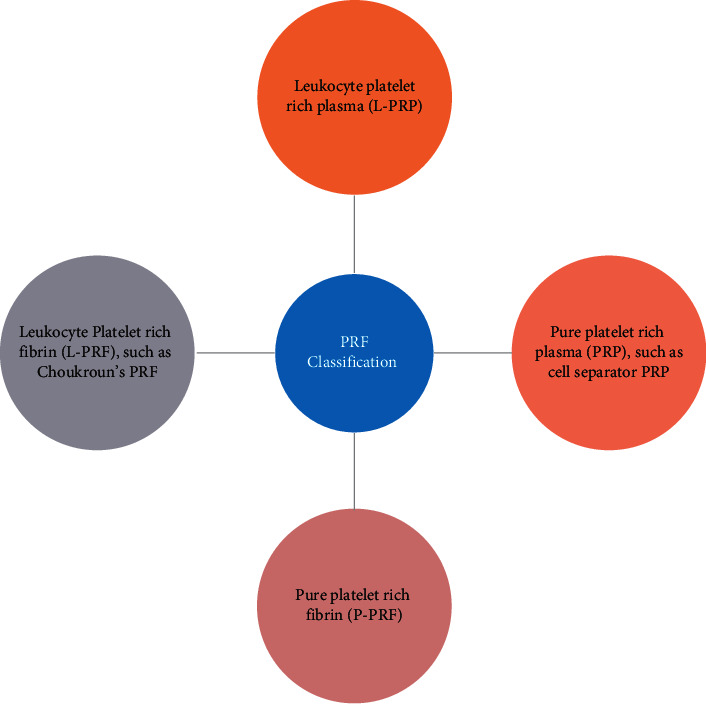
Classification based on leukocytes.

Initially, platelet concentrates in transfusion medicine were intended to treat and prevent hemorrhage due to various conditions. Several years ago, blood-derived products were used to seal wounds and promote healing using fibrin glues. These fibrin glues consisted of concentrated fibrinogen [6]. Autologous origin reduces the risk of contamination [7]. Consequently, further exploration in the field led to the replacement of fibrin glue with platelet concentrates, which was first described by Whitman et al. [8]. Consequently, platelet-rich fibrin (PRF) as a tool for tissue regeneration in medicine was introduced in 2001 [9]. The concept was extrapolated from the first-generation platelet concentrate, i.e., platelet-rich plasma (PRP). Its efficacy in various medical fields was astounding despite the setback of inhibiting the coagulation cascade because of an anticoagulant to the preparation [10–12]. With further advancement, Leukocyte PRF (L-PRF) came into being. It is named as such because of the higher leukocyte count. L-PRF acts as a three-dimensional fibrin matrix that entraps growth factors, with the added benefit of the absence of anticoagulants to the formulation [13–15]. A classification was proposed based on leukocyte and fibrin content in which platelet concentrates were categorized into four categories [16], as shown in Figure 2.

### 3.1. Leucocyte-Poor or Pure Platelet-Rich Plasma (P-PRP)

Pure platelet concentrates were first developed for topical use as a supplemental application to the classical platelet units, and their first clinical implementation was reported in maxillofacial surgery [8, 17].

The method of producing platelet concentrates for topical use is called plasmapheresis, which requires a cell separator that separates the blood into different components such as platelets, leukocytes, and erythrocytes, which can then be used and readministered to the patient [18].

### 3.2. Leucocyte- and Platelet-Rich Plasma (L-PRP)

The primary goal of developing an alternative, more convenient method was to incorporate platelet concentrates in day-to-day practice without the need for a transfusion laboratory. Initially, the product collected had high quantities of leucocytes which were hard to eliminate without the help of a cell separator. However, the alteration in collection parameters made way for pure (leucocyte free) PRP to be collected through more rigorous experimentation. The main setback of this technique is the essential requirement of expensive and complex centrifuges and preparation kits. Furthermore, like fibrin glue, the final product dissolves quickly. Consequently, L-PRP is rendered uncommon for daily use [19].

### 3.3. Leucocyte-Poor or Pure Platelet-Rich Fibrin (P-PRF) Concentrates

To produce pure platelet-rich fibrin, a small quantity of blood is collected into a collection tube. Trisodium citrate is added as an anticoagulant and a separator gel, and the suspension is centrifuged at high speed for six minutes. The separated buffy coat and platelet-poor plasma (PPP) are transferred into another tube containing calcium chloride and centrifuged. The stable clot is collected and being used.

The company that developed this method claimed that a “natural” platelet formulation is produced since bovine thrombin was not added. However, these claims are questionable because synthetic compounds are involved in the process (i.e., anticoagulant and separator gel) [19].

### 3.4. Leucocyte- and Platelet-Rich Fibrin (L-PRF) Concentrates

Choukroun et al. developed a simple technique for producing L-PRF in France [20]. Venous blood is drawn into glass tubes and centrifuged at a low speed [21]. Platelet activation and polymerization of fibrin are instantaneous due to the absence of anticoagulants. The PRF clot produced has several clinical applications in oral [22], maxillofacial surgery [23, 24], ENT [25], and plastic surgery [26]. This preparation has the advantage of gradual dissolution after application, and the three-dimensional fibrin mesh slowly remodels, corresponding to the physiological blood clot.

Moreover, the technique is simple and efficient; higher quantities are obtainable, involving only natural constituents as reactants. Thus, L-PRF is most appropriate for everyday practices, and many countries such as France, Israel, and Italy have already employed it [19].

## 4. Mechanism of Action of Platelet-Rich Fibrin

**Figure 3:**
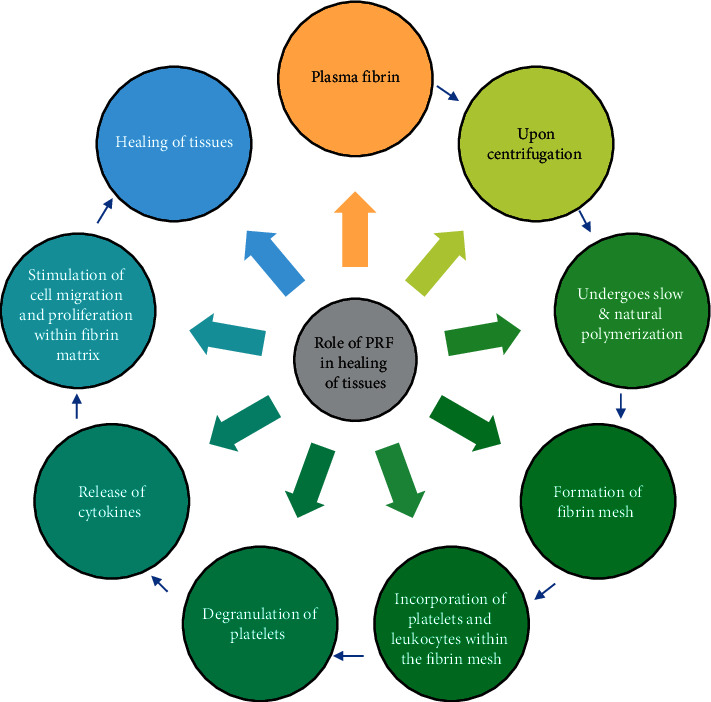
Role of PRF in healing of tissues.

PRF consists of a packed fibrin complex consisting of leukocytes, cytokines, and glycoproteins such as thrombospondin. In a condensed PRF scaffold, leukocytes hold an integral position in growth factor release in addition to an immune response. Promotion of tissue regeneration and wound healing is achieved due to this concentrated suspension of platelets rich in growth factors. Transforming growth factor beta (TGF-*β*) accelerates reactionary dentinogenesis by stimulating odontoblastic activity [27]. Leukocytes, cytokines, and lymphocytes inhibit infection and inflammatory cascades. Vascular endothelial growth factor (VEGF) aids in angiogenesis which is pivotal in revascularization [28], Figure 3.

### 4.1. Role of Fibrin in Angiogenesis

Cytokines including FGF, VEGF, angiopoietin, and PDGF get entrapped into the fibrin matrix's three-dimensional structure, resulting in a slowly progressing release, which is imperative in angiogenesis [29]. Fibrin causes the enhanced expression of *αvβ*3 integrin which stimulates the binding of endothelial cells to fibrin, fibronectin, and vitronectin [30].

### 4.2. Fibrin-Assisted Immune Response

Adhesion to endothelial cells, fibrinogen, and the transmigration of neutrophils is aided by fibrin. Fibrin acts by heightening the expression of CD11c/CD18 receptors on endothelial cells [31]. The wound colonization by macrophages is modulated by fibrin and fibronectin.

### 4.3. Effect of Fibrin on Mesenchymal Stem Cells

The fibrin matrix provides a scaffold for undifferentiated mesenchymal cells and promotes differentiation, imperative for tissue regeneration [22].

### 4.4. Effect of Fibrin on Osseous Tissue

Similarly, fibrin also acts as a scaffold for bone morphogenic protein, and its sustained release from within the fibrin matrix induces bone formation. Steady release of VEGF, FGF, and PDGF promotes angiogenesis. The circulating stem cells get entrapped into the fibrin clot resulting in hemostasis, thus enabling tissue restoration [32].

## 5. PRF in Dentistry

The usage of PRF has reported high success rates in surgical cases such as sinus lift procedures. Bone height and width are maintained, and wound healing is also achieved. In cases where immediate implants are to be placed, PRF has been reported to play a significant role in rapid healing of the extraction socket PRF, along with bone graft, and is known to give a synergetic effect. Intrabony defects are managed with open flap debridement and PRF to gain the clinical attachment loss [33]. According to a case report, an avulsed tooth with a periapical abscess was treated by shaping the canal, and a triple antibiotic paste was placed until the follow-up. In return, the antibiotic paste was removed, and the canal was irrigated. Revascularization was performed, and PRF was prepared and inserted in the canal, followed by biodentine and glass ionomer cement placement. After 6 months of follow-up, PRF helped in the apex's closure and repair and thickening of radicular dentin [34].

### 5.1. Platelet-Rich Fibrin in Regenerative Endodontics

Numerous conditions require endodontic intervention, including dental caries and pulpitis, prevalent in more than two-thirds of the world's population [35]. Additionally, dental trauma amongst children results in pulpal tissue damage, with concerns mainly in the immature tooth, as treatment options are limited in such cases of open apices [36]. However, with the advent of the treatment modality of revascularization, the tooth's survival has increased. It has positively impacted the management of symptoms, and postoperative radiographs confirm the physiological root completion [37].

Traditionally, revascularization compromised the induction of a blood clot into the root canal space [38], which emerged as a clinical triumph [39]. This further led to platelet concentrates as an autologous scaffold on which revascularization could occur [40]. The applications of platelet-rich fibrin (PRF) in regenerative endodontics are numerous. Bains et al. employed it as the agent for repairing iatrogenic perforation of the pulpal floor of the mandibular first molar when used in combination with MTA [41]. PRF is ideal for the revascularization of immature permanent teeth with necrotic pulps by providing a scaffold rich in growth factors, enhancing cellular proliferation and differentiation. It acts as a matrix for tissue ingrowth [42]. Furthermore, the gradual release of growth factors as the fibrin matrix resorbs ensures a steady healing process [43]. Evidence of progressive thickening of dentinal walls, root lengthening, regression in the periapical lesion, and apical closure was reported by Shivashankar et al., following the use of PRF on a tooth with pulpal necrosis and open apex [44].

Similarly, successful healing and apexification with the combined use of MTA as an apical barrier and autologous platelet-rich fibrin membrane as an internal matrix were reported by Rudagi K. and B. Rugadi. [45]. Additionally, PRF enhanced dental pulp cell proliferation, upregulation in alkaline phosphatase activity, and increased osteoprotegerin expression in a time-dependent fashion [46]. Pulpotomy in young permanent teeth using PRF has been reported to have affirmative results [47]. Moreover, instead of solely using biomaterials for the bone augmentation following treatment of periapical defects, the combination of PRF with a biomaterial (*β*-TCP) offers a better treatment alternative for swift healing [48]. It exhibits a more predictable radiographic and clinical bony regeneration [49].

Cases of traumatic immature teeth with necrotic pulp were treated with calcium hydroxide that induced a calcific barrier, but it reduces the organic support of dentin (radicular), which ceases root fracture. In order to prevent the chances of fracture, platelet-rich fibrin serves to be the best alternative that maintains tooth vitality and strength [42].

## 6. Clinical Implementation

### 6.1. PRF in Oral and Maxillofacial Surgery (OMFS)

The management of dimensional changes of the alveolar bone directly following tooth extraction has become an area of interest amongst researchers [50]. A study of 23 patients showed that the use of PRF resulted in reduced changes in dimension of the alveolar bone before implant placement in comparison to natural socket healing [51]. Furthermore, it was seen that there was nearly a ten-fold decrease in the rate of osteomyelitis infections when the third molar extraction sockets were filled using PRF [52]. Additionally, there are multiple other uses of PRF in oral and maxillofacial surgery.

PRF is routinely utilized in sinus lift procedures, particularly as a filling material during sinus lift and implant surgeries [53]. Several studies have proven the efficacy of L-PRF in new bone formation and its role in wound healing. Procedures such as alveolar ridge augmentation involve the use of PRF-based membranes [54]. Preliminary positive outcomes were observed when such membranes were used in patients on anticoagulant therapy and the prevention and treatment of patients on bisphosphonate-induced osteonecrosis of the jaw. L-PRF can be used to fill up cavities after tumor removal [55] and should be further investigated as a regenerative material after excision of malignant tumor tissue. It can be used in plastic reconstructive surgeries as an adjunct with graft material (adipocytes) [26]. Further experimentation and understanding of the appropriate surgical methodology are required in other fields of OMFS, such as orthognathic surgery (Lefort osteotomies, etc.), to benefit from L-PRF properties [56].

### 6.2. PRF in Periodontics

PRF is used in the regeneration of the periodontium. It is enriched with soluble growth factors and cytokines, including TGF-*β*1, VEGF, ILGF, PDGF, and Il-1, -4, and -6, which mainly aid tissue regeneration and accelerate wound healing [57]. There is a notable improvement in the clinical outcome and the radiographic reduction in the intrabony defect depth when either bone grafts or pharmacological agents such as metformin gel is used in combination with PRF of being used alone [57]. Similarly, investigations of periodontal regeneration of class II furcation defects using PRF were studied. According to this, there was a significant improvement in clinical attachment loss (CAL) gains with the use of PRF when compared to open flap debridement (OFP) [58–60]. In conclusion, these results signify the tissue repair potential using PRF for furcation defects.

According to a study carried out, during the coverage of gingival recession by raising a coronal advancement flap, the addition of PRF to the area showed a reduction in matrix metalloproteinase 8 and IL beta levels initially, and an increase in matrix metalloproteinase 1 level at day 10 was observed. This resulted in promoting periodontal wound healing in the earlier phase of the process [57].

### 6.3. Future Recommendation

To maintain bone height procedures in avulsion and cystic excision cases where implants are placed, we need to use platelet-rich fibrin. Compared to platelet-rich plasma (PRP), platelet-rich fibrin (PRF) is more economical, easy to prepare, and feasible to use in daily clinical practices. In periodontal procedures where guided tissue regeneration (GTR) is performed to overcome intrabony defects, platelet-rich fibrin (PRF) membranes are expected to be replaced. They are compatible with patients who have diabetes, who smoke, or who use anticoagulants, while using platelet-rich plasma (PRP) cannot be facilitated. Fibrin membrane acts as a natural stimulating barrier and prevents soft- and hard-tissue integration, promoting bone regeneration and filling the defect [61]. Studies are needed to clarify the precise mechanism of action of PRF for dental pulp regeneration both in vitro and in vivo.

## Data Availability

All data used to support the findings of this study are included in the article.
